# Impact of incisura biopsy on the surveillance of precursor lesions and gastric cancer risk assessment

**DOI:** 10.1093/gastro/goag055

**Published:** 2026-05-29

**Authors:** Elisa Cantú-Germano, Sonia Torres, Glòria Fernández-Esparrach, Miriam Cuatrecasas, Sandra López-Prades, Irina Luzko, Anabella Cuestas, Joaquín Castillo-Iturra, Francesc Balaguer, Leticia Moreira

**Affiliations:** Servei de Gastroenterologia, Hospital Clínic de Barcelona, Universitat de Barcelona (UB), 08036 Barcelona, Catalunya, Spain; Institut d’Investigacions Biomèdiques August Pi i Sunyer (IDIBAPS), 08036 Barcelona, Catalunya, Spain; Facultat de Medicina i Ciències de la Salut, Universitat de Barcelona (UB), 08036 Barcelona, Catalunya, Spain; Servei de Gastroenterologia, Hospital Clínic de Barcelona, Universitat de Barcelona (UB), 08036 Barcelona, Catalunya, Spain; Servei de Gastroenterologia, Hospital Clínic de Barcelona, Universitat de Barcelona (UB), 08036 Barcelona, Catalunya, Spain; Institut d’Investigacions Biomèdiques August Pi i Sunyer (IDIBAPS), 08036 Barcelona, Catalunya, Spain; Facultat de Medicina i Ciències de la Salut, Universitat de Barcelona (UB), 08036 Barcelona, Catalunya, Spain; Centro de Investigación Biomédica en Red de Enfermedades Hepáticas y Digestivas (CIBEREHD), Instituto de Salud Carlos III, 28029 Madrid, Madrid, Spain; Institut d’Investigacions Biomèdiques August Pi i Sunyer (IDIBAPS), 08036 Barcelona, Catalunya, Spain; Facultat de Medicina i Ciències de la Salut, Universitat de Barcelona (UB), 08036 Barcelona, Catalunya, Spain; Centro de Investigación Biomédica en Red de Enfermedades Hepáticas y Digestivas (CIBEREHD), Instituto de Salud Carlos III, 28029 Madrid, Madrid, Spain; Servei de Patologia, Hospital Clínic de Barcelona, Universitat de Barcelona (UB), 08036 Barcelona, Catalunya, Spain; Institut d’Investigacions Biomèdiques August Pi i Sunyer (IDIBAPS), 08036 Barcelona, Catalunya, Spain; Servei de Patologia, Hospital Clínic de Barcelona, Universitat de Barcelona (UB), 08036 Barcelona, Catalunya, Spain; Servei de Gastroenterologia, Hospital Clínic de Barcelona, Universitat de Barcelona (UB), 08036 Barcelona, Catalunya, Spain; Institut d’Investigacions Biomèdiques August Pi i Sunyer (IDIBAPS), 08036 Barcelona, Catalunya, Spain; Facultat de Medicina i Ciències de la Salut, Universitat de Barcelona (UB), 08036 Barcelona, Catalunya, Spain; Centro de Investigación Biomédica en Red de Enfermedades Hepáticas y Digestivas (CIBEREHD), Instituto de Salud Carlos III, 28029 Madrid, Madrid, Spain; Servei de Gastroenterologia, Hospital Clínic de Barcelona, Universitat de Barcelona (UB), 08036 Barcelona, Catalunya, Spain; Institut d’Investigacions Biomèdiques August Pi i Sunyer (IDIBAPS), 08036 Barcelona, Catalunya, Spain; Facultat de Medicina i Ciències de la Salut, Universitat de Barcelona (UB), 08036 Barcelona, Catalunya, Spain; Servei de Gastroenterologia, Hospital Clínic de Barcelona, Universitat de Barcelona (UB), 08036 Barcelona, Catalunya, Spain; Institut d’Investigacions Biomèdiques August Pi i Sunyer (IDIBAPS), 08036 Barcelona, Catalunya, Spain; Facultat de Medicina i Ciències de la Salut, Universitat de Barcelona (UB), 08036 Barcelona, Catalunya, Spain; Servei de Gastroenterologia, Hospital Clínic de Barcelona, Universitat de Barcelona (UB), 08036 Barcelona, Catalunya, Spain; Institut d’Investigacions Biomèdiques August Pi i Sunyer (IDIBAPS), 08036 Barcelona, Catalunya, Spain; Facultat de Medicina i Ciències de la Salut, Universitat de Barcelona (UB), 08036 Barcelona, Catalunya, Spain; Centro de Investigación Biomédica en Red de Enfermedades Hepáticas y Digestivas (CIBEREHD), Instituto de Salud Carlos III, 28029 Madrid, Madrid, Spain; Servei de Gastroenterologia, Hospital Clínic de Barcelona, Universitat de Barcelona (UB), 08036 Barcelona, Catalunya, Spain; Institut d’Investigacions Biomèdiques August Pi i Sunyer (IDIBAPS), 08036 Barcelona, Catalunya, Spain; Facultat de Medicina i Ciències de la Salut, Universitat de Barcelona (UB), 08036 Barcelona, Catalunya, Spain; Centro de Investigación Biomédica en Red de Enfermedades Hepáticas y Digestivas (CIBEREHD), Instituto de Salud Carlos III, 28029 Madrid, Madrid, Spain

**Keywords:** atrophic gastritis, biopsy, incisura, intestinal metaplasia, precancerous conditions, stomach neoplasms

## Abstract

**Background:**

The diagnostic value of an incisura biopsy during upper gastrointestinal endoscopies (UGEs) for detecting gastric cancer precursor lesions (GCPLs) remains debated. This study aimed to evaluate the impact of incisura biopsy on the detection of GCPL, staging according to Operative Link for Gastritis Assessment (OLGA) and Operative Link of Gastritis and Intestinal Metaplasia (OLGIM), and surveillance strategies.

**Methods:**

This observational, single-centre retrospective study included patients undergoing UGE between 2017 and 2022 with gastric biopsies from the corpus, antrum, and incisura in separate vials, and intestinal metaplasia (IM) identified in at least one site. OLGA/OLGIM stages were assessed with and without the incisura biopsy. Factors associated with stage or surveillance modifications were analysed.

**Results:**

Among 27,166 patients undergoing UGEs, 819 patients met the inclusion criteria. Of the 819 patients, the median age was 67 years (interquartile range, 59–74 years), 59.5% were women, 89.3% originated from low gastric cancer (GC) risk regions, and 35.6% had current or prior *Helicobacter pylori* infection. The incisura presented isolated atrophy, IM, and dysplasia in 5.9%, 11.1%, and 1.6%, respectively. Its inclusion upstaged OLGA/OLGIM in 18.3% and 16.5%, respectively, which led to surveillance recommendations in 7.0% and 2.6% patients, respectively. Overall, this corresponded to a 9.0% absolute increase in patients recommended for surveillance compared with omitting the incisura biopsy. OLGIM upstage was associated with current *H. pylori* infection, alcohol intake, and origin from an intermediate GC-risk region, whereas surveillance changes were associated only with intermediate-risk origin.

**Conclusions:**

Adding an incisura biopsy to gastric mapping identified more advanced GCPL, upstaged OLGA/OLGIM in ∼20% of patients, and increased surveillance recommendations in an extra ∼10%. These results recommend the systematic inclusion of the incisura biopsy in GC risk stratification.

## Introduction

Gastric cancer (GC) is the fifth most common malignancy worldwide and the third leading cause of cancer-related mortality [1–3]. Its incidence varies geographically, with higher rates in Asia and lower rates in Europe [2, 4]. Gastric intestinal-type adenocarcinoma develops through the Pelayo-Correa Cascade, a multistep process in which chronic atrophic gastritis, intestinal metaplasia (IM), and dysplasia are recognized as GC precursor lesions (GCPL) [3, 4].

The risk of progression to GC increases with both the extent and severity of GCPL, with estimated annual progression rates of 0.1% for atrophic gastritis, 0.25% for IM, and 0.6%–6% for dysplasia [5]. To stratify GCPL and estimate GC risk, the Operative Link for Gastritis Assessment (OLGA) and Operative Link of Gastritis and Intestinal Metaplasia (OLGIM) classifications are commonly used [1, 4–6]. These systems rely on histological evaluation of gastric biopsies obtained according to the updated Sydney protocol, which recommends five samples: two from the antrum, two from the corpus, and one from the incisura [7].

However, the utility of the incisura biopsy has been recently debated. Previous studies suggested that omitting the incisura biopsy may lead to underestimation of GC risk [1, 8, 9]. In contrast, the recent MAPS III guideline states that biopsy of the incisura is optional and should be performed at the endoscopist’s discretion [1, 4]. This guideline currently recommends endoscopic surveillance for patients with high-risk GCPL, defined as those with OLGA or OLGIM stages III–IV, or lower stages combined with family history of GC or persistent *Helicobacter pylori (H. pylori)* infection [1].

The present study aimed to evaluate the role of incisura biopsy in the detection of advanced GCPL and in GC risk stratification by comparing OLGA and OLGIM classifications with and without the inclusion of the incisura biopsy, as well as to assess the potential impact of the incisura biopsy on endoscopic surveillance recommendations for patients with GCPL.

## Methods

### Patients and study design

This observational, retrospective, single-centre study included the patients who underwent upper gastrointestinal endoscopy (UGE) at the Endoscopy Unit of Hospital Clinic of Barcelona, Spain between January 2017 and December 2022. UGEs performed in patients aged over 18 years with gastric biopsies were reviewed.

To evaluate the impact of incisura biopsy on GC risk stratification by using the OLGA and OLGIM systems, the cohort was intentionally enriched with patients with histologically confirmed IM. This strategy allowed a focused assessment of the role of incisura biopsy in patients with GCPL, rather than evaluating its diagnostic yield in an unselected endoscopic population. Inclusion criteria required the availability of gastric biopsies obtained from the corpus, antrum and incisura, with specimens placed in separate vials. Only cases presenting IM in at least one gastric biopsy were included. Exclusion criteria were subsequent UGEs performed on the same patient and incomplete UGEs.

The study was approved by the Ethics Committee of the Hospital Clinic of Barcelona (HCB/2021/0482; 20 April 2022) and conducted in accordance with the principles of the Declaration of Helsinki.

### Endoscopic assessment

The UGEs were performed by experienced endoscopists at a tertiary centre using high-definition endoscopes according to routine clinical practice in the study period. Image-enhanced endoscopy techniques, including virtual chromoendoscopy, were used at the discretion of the endoscopist.

Gastric biopsies were performed by using standard biopsy forceps, obtained following the updated Sydney protocol. The incisura biopsy was obtained from the incisura angularis along the lesser curvature, corresponding to the transitional zone between the antrum and the corpus. Biopsies from each anatomical site were placed in separate vials.

### Pathological assessment

Gastric biopsy specimens were processed and evaluated by experienced gastrointestinal pathologists following standard pathological protocols (using haematoxylin & eosin–stained sections, and additional stains when pathologically needed). Biopsy samples from each anatomical site were examined separately. The pathological report included both the presence and severity of gastric atrophy and IM using qualitative categories (absent, mild, moderate, or severe), based on the pathologists’ estimation of the proportion of gastric mucosa affected by each GCPL. Then the OLGIM stage was stated on the pathological report.

### Data collection

The registry included clinical, endoscopic, and histopathological variables. Clinical variables recorded were age, sex, country of origin, alcohol and tobacco exposure, UGE indication, previous *H. pylori* infection, and eradication. Endoscopic features included the use of high-definition endoscopy and chromoendoscopy, as well as the presence of visible lesions in the incisura. Histopathological variables included in the pathological report described the presence and severity of gastric atrophy and IM at each biopsy site, as well as the presence or absence of *H. pylori* in the current UGE.

### Variable categorization and definition

The ‘UGE indication’ was reclassified into the following categories: surveillance of high-risk conditions of GC (including GCPL, family or personal history of GC, *CDH1* gene mutation, polyposis syndromes), symptoms, follow-up of digestive diseases, and other indications.

‘GC risk by country of origin’ was defined based on age-adjusted incidence of GC. Countries were categorized as high-risk if the annual incidence exceeded 20 cases per 100,000 population, intermediate when 10–20 cases per 100,000 people, and low-risk if below 10 cases per 100,000 inhabitants [10].

For the purposes of this study, the severity of gastric atrophy and IM reported for each biopsy site was extracted from the pathological report. The OLGIM stage was already reported by the pathologists. The OLGA stage was calculated by gastroenterologists using the pathological information provided in the pathological report. The qualitative categories were subsequently converted into semi-quantitative scores (absent = 0, mild = 1, moderate = 2, severe = 3), according to the distribution and severity of atrophy and IM across the sampled gastric regions. This was defined as a practice-oriented tool rather than a formally validated system and called in this study as modified OLGA stage (mOLGA), although it corresponded the conventional OLGA stage allowing its calculation. Finally, to evaluate the specific contribution of the incisura biopsy, both mOLGA and OLGIM stages were calculated with and without inclusion of the incisura specimen.

### Statistical analysis

Continuous variables are presented as medians with interquartile ranges (25th and 75th percentiles). Qualitative variables are presented as absolute and relative frequencies, expressed as percentages.

Associations between categorical variables were assessed by using chi-square test. Statistical significance was defined as *P* value ˂0.05. Descriptive and univariate analyses included sex, age, GC risk by country of origin (low/medium/high), tobacco and alcohol consumption (absent/previous/current), UGE indication, *H. pylori* status (current or past), presence of visible lesions in incisura, use of chromoendoscopy, and high-resolution endoscopy.

Multivariate analysis was performed with logistic regression, including variables that showed statistical significance (*P *< 0.05) in the univariate analysis. The effect was evaluated by calculating odds ratios (ORs) with corresponding 95% confidence intervals (CIs). All statistical tests were two‐sided. Analyses were performed using SPSS version 29 (IBM).

## Results

### Baseline characteristics

During the study period of time, 27,166 patients underwent UGE with biopsies and 1,440 had IM detected in at least one gastric biopsy. Among these, 819 patients had gastric biopsies according to the updated Sydney protocol, allocated in separate vials and were included in the final analysis (Supplementary Fig. S1).

Among the 819 included patients, 59.5% were women, and the median age was 67 years (59–74 years). Most patients were from Spain (82.3%), followed by Peru (3.2%), Ecuador (2.4%), China (2.2%), and Colombia (1.8%); the remaining patients originated from 29 other countries. Regarding GC risk based on country of origin, 89.2% of patients were from low-risk regions, 10.4% from intermediate-risk, and 0.4% from high-risk regions (Table 1).

**Table 1:** Baseline characteristics of 819 patients

Characteristic	No. of patients (%)
Sex	
Male	332 (40.5%)
Female	487 (59.5%)
Age (years), median (IQR)	67 (59–74)
GC risk by country of origin	
Low	731 (89.3%)
Intermediate	85 (10.3%)
High	3 (0.4%)
Smoking habit	
Absent	469 (57.3%)
Previous exposure	210 (25.6%)
Current exposure	139 (16.9%)
Alcohol consumption	
Absent	697 (85.1%)
Previous exposure	32 (3.9%)
Current consumption	89 (10.9%)
UGE indication	
Symptoms	399 (48.7%)
Surveillance of high-risk of GC	295 (36.1%)
Digestive disease	96 (11.7%)
Others	29 (3.5%)
*H. pylori* on current UGE	127 (15.5%)
Previous *H. pylori*^a^	165/526 (31.4%)
Overall prevalence of *H. pylori*	292 (35.6%)
Visible lesions in incisura	73 (8.9%)
Intestinal metaplasia	44 (5.4%)
Erosions	10 (1.2%)
Ulcers	9 (1.1%)
Polyps	2 (0.2%)
Scars	4 (0.5%)
Inflammatory changes	4 (0.5%)
Chromoendoscopy	230 (28.1%)
High-definition endoscopy	819 (100%)

a Only applicable in cases without *H. pylori* in the current UGE.

IQR, interquartile range; GC, gastric cancer; UGE, upper gastrointestinal endoscopy.

Regarding toxic habits, 16.9% were current smokers, and 25.6% former smokers. Daily alcohol consumption was reported in 10.9%, while 3.9% reported previous alcohol use. The main indications for UGE were symptoms (48.7%) and surveillance of GC high-risk conditions (36.1%) (Table 1).

### Endoscopic assessment

All UGEs were performed by using high-definition gastroscopes, which represented the standard practice of the centre during the study period. Virtual chromoendoscopy was employed at the endoscopists’ discretion in 28.1% of UGEs.

**Figure 1:**
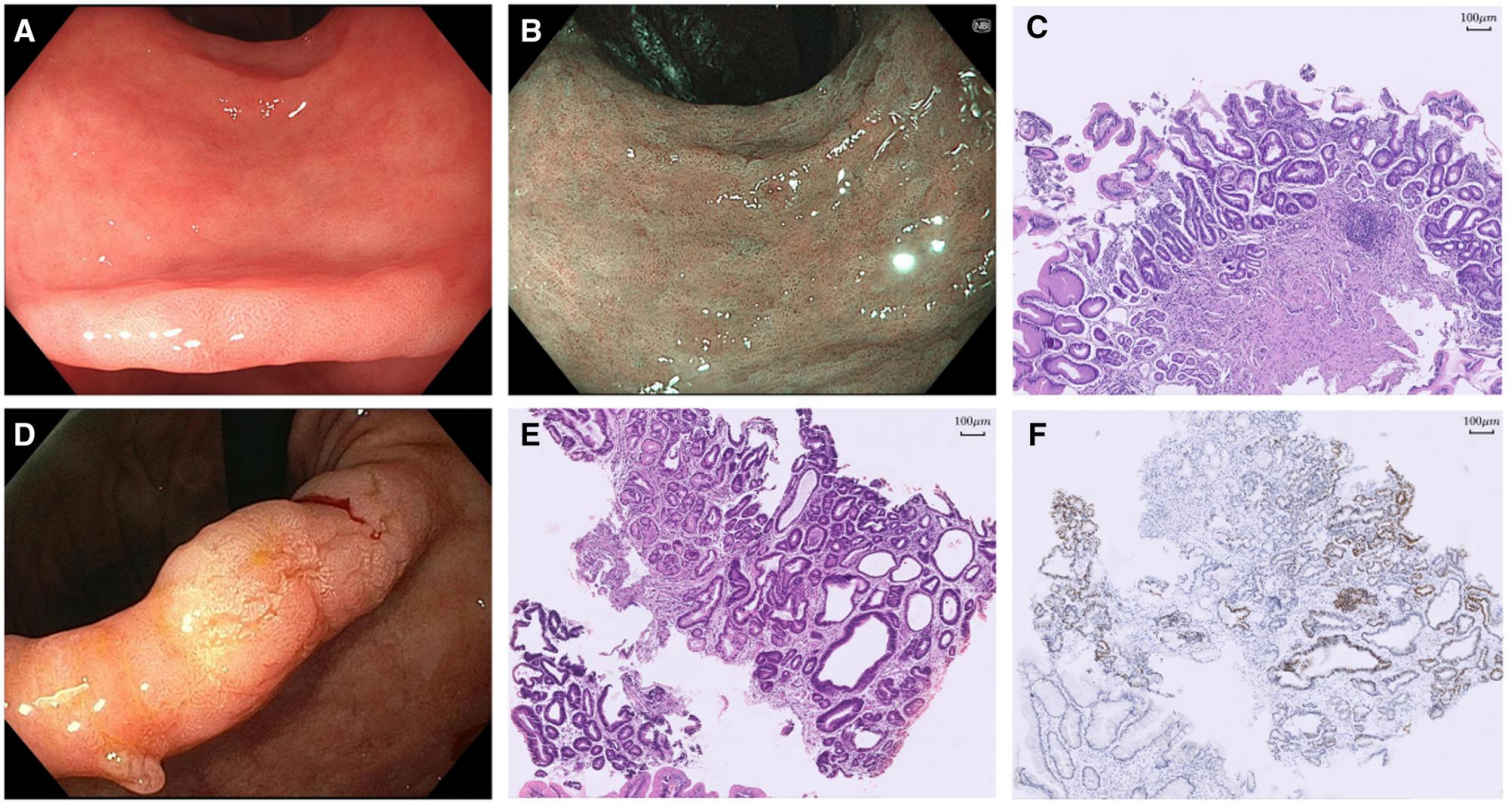
Representative endoscopic findings of IM and dysplasia in incisura and the corresponding pathological assessment. (A) White light and (B) narrow band imaging endoscopic assessment of the incisura shows atrophic mucosa with extensive areas of intestinal metaplasia. (C) Microscopic view of endoscopic biopsy from case A/B shows moderate chronic atrophic gastritis with moderate intestinal metaplasia (haematoxylin & eosin stain, ×10). (D) White light endoscopic assessment of the incisura shows slightly elevated and irregular areas with pale colouration. (E) Microscopic view of endoscopic biopsy from case D shows moderate chronic atrophic gastritis with intestinal metaplasia and superficial *foci* of high-grade dysplasia (haematoxylin & eosinstain, ×10). (F) p53 immunostaining highlights superficial *foci* of high-grade dysplasia (p53, ×10).

Focusing on the incisura, a visible lesion was reported in 8.9% (*n *= 73), of which 60.3% (44/73) were described endoscopically as IM (Table 1). Figure 1 illustrates representative endoscopic findings of IM and dysplasia in incisura, along with the corresponding pathological assessment.

### Pathological findings

Gastric atrophy was identified in 90.6% (742/819) of patients. According to biopsy location, atrophy was present in 53.8% (441/819) of corpus, 70.5% (577/819) of antrum, and 70.9% (581/819) of incisura biopsies. The incisura represented the only involved site in 6.6% (49/742) of patients with atrophy. Consequently, not performing the incisura biopsy would have resulted in missed diagnosis of gastric atrophy in 5.9% (49/819) of patients.

IM was present in all patients, as its presence in at least one biopsy site was an inclusion criterion. According to gastric topography, IM was identified in 47.9% (393/819) of corpus, 62.1% (509/819) of antrum, and 61.4% (503/819) of incisura biopsies. The incisura was the only site with IM in 11.1% (91/819) of patients; therefore, these cases would have been missed if incisura biopsy had not been performed.

Dysplasia was identified in 4.5% (37/819) of patients. It was detected in 0.9% (8/819) of corpus, 2.1% (17/819) of antrum, and 2.3% (19/819) of incisura biopsies. In 35.1% (13/37), the incisura was the only site harbouring dysplasia. Therefore, a diagnosis of dysplasia would have been missed in 1.6% (13/819) of the patients if the incisura biopsy had been omitted.

### *H. pylori* status

Active *H. pylori* infection was identified by pathological assessment in 15.5% (127/819) of patients. Among the 692 patients without active infection, previous information regarding *H. pylori* status was available for 526 individuals, of whom 31.4% (165/526) had a documented history of prior infection. When considering both current and previous *H. pylori* infection, the overall prevalence was 35.6% (292/819).

### mOLGA staging

**Figure 2:**
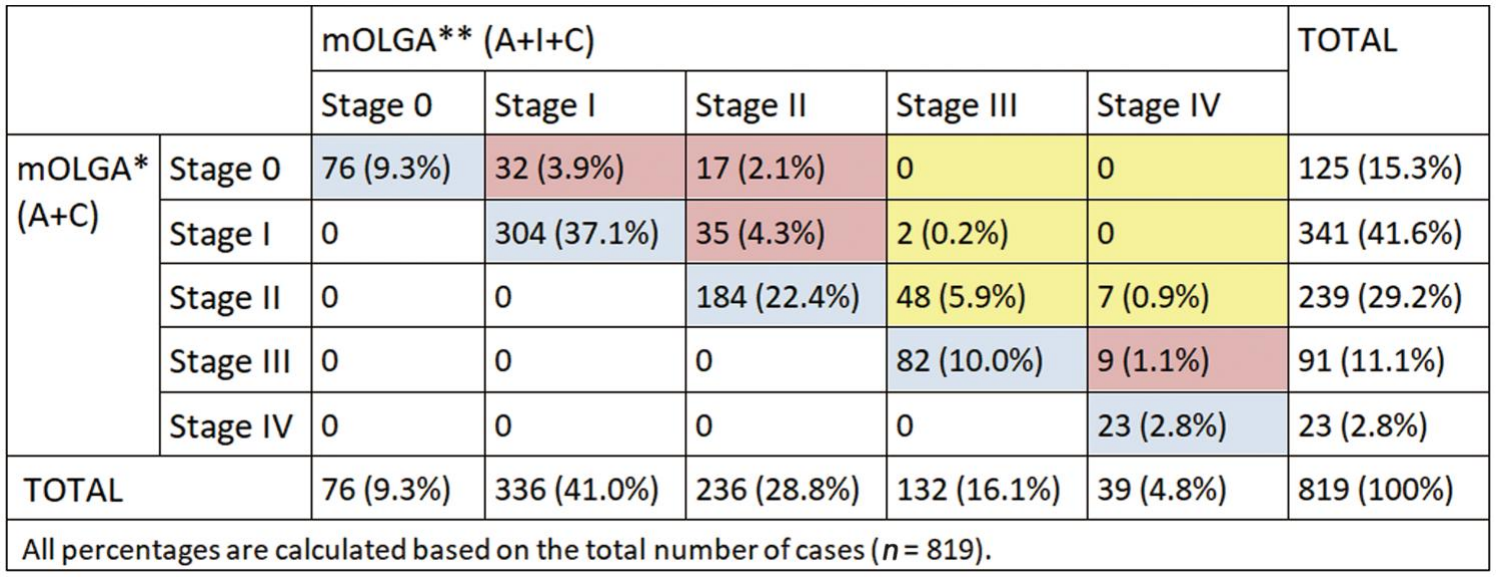
Comparison of mOLGA stage between using only biopsies from the antrun and the corpus and when adding incisura biopsy. *mOLGA using only antrum and corpus biopsies. **mOLGA using antrum, incisura, and corpus biopsies. Pink indicates mOLGA upstaging only. Yellow indicates mOLGA upstaging that led to endoscopic surveillance recommendation. Blue indicates no changes in mOLGA stage when adding the incisura biopsy. mOLGA = modified Operative Link for Gastritis Assessment, A = antrum, I = incisura, C = corpus.

The mOLGA stage was calculated twice: first using biopsies from the corpus and antrum only, and then by adding the incisura biopsy. Without the incisura biopsy, the distribution of mOLGA stages was 15.3% stage 0, 41.6% stage I, 29.2% stage II, 11.1% stage III, and 2.8% stage IV. After including the incisura biopsy, the distribution shifted to 9.3% stage 0, 41.0% stage I, 28.8% stage II, 16.1% stage III, and 4.8% stage IV. Consequently, the proportion of patients classified as high-risk stages (III–IV) increased from 13.9% to 20.9% (a 7.0% increase). Overall, incorporating the incisura biopsy upstaged 18.4% of cases (Fig. 2). Among patients with mOLGA upstaging, 44.7% also presented concurrent OLGIM upstaging. No clinical or endoscopic factors were significantly associated with mOLGA upstaging (Table 2).

**Table 2:** Associated factors to change in mOLGA stage and follow-up of gastric atrophy—univariate analysis

Variable	mOLGA stage			Follow-up of gastric atrophy		
	No change (*N *= 669)	Upstage (*N *= 150)	*P* value	No change (*N *= 762)	Change in follow-up (*N *= 57)	*P* value
Sex, *n* (%)			0.339			0.276
Male	266 (39.8%)	66 (44.0%)		305 (40.0%)	27 (47.4%)	
Female	403 (60.2%)	84 (56.0%)		457 (60.0%)	30 (52.6%)	
Age, *n* (%)			0.054			0.335
<67 years	312 (46.6%)	83 (55.3%)		364 (47.8%)	31 (54.4%)	
≥ 67 years	357 (53.4%)	67 (44.7%)		398 (52.2%)	26 (45.6%)	
GC risk by country of origin, *n* (%)			0.527			0.817
Low	594 (88.8%)	137 (91.3%)		679 (89.1%)	52 (91.2%)	
Intermediate	72 (10.8%)	13 (8.7%)		80 (10.5%)	5 (8.8%)	
High	3 (0.4%)	0 (0%)		3 (0.4%)	0 (0%)	
Smoking habit, *n* (%)			0.730			0.978
Absent	387 (57.8%)	82 (54.7%)		436 (57.2%)	33 (57.9%)	
Previous exposure	168 (25.1%)	42 (28.0%)		196 (25.7%)	14 (24.6%)	
Current exposure	113 (16.9%)	26 (17.3%)		129 (16.9%)	10 (17.5%)	
Alcohol consumption, *n* (%)						
Absent	575 (85.9%)	122 (81.3%)	0.253	646 (84.7%)	51 (89.5%)	0.581
Previous exposure	26 (3.9%)	6 (4.0%)		31 (4.1%)	1 (1.7%)	
Current consumption	67 (10.0%)	22 (14.7%)		84 (11.0%)	5 (8.8%)	
Chromoendoscopy, *n* (%)	193 (28.8%)	37 (24.7%)	0.303	218 (28.6%)	12 (21.1%)	0.221
High-definition endoscopy, *n* (%)	669 (100%)	150 (100%)	–	762 (100%)	57 (100%)	–
Current *H. pylori* infection, *n* (%)	103 (15.4%)	24 (16.0%)	0.853	120 (15.7%)	7 (12.3%)	0.485
Previous *H. pylori*,^a^ *n* (%)	134 (20.3%)	31 (20.7%)	0.769	156 (20.5%)	9 (15.8%)	0.456
Visible lesion in incisura, *n* (%)	59 (8.8%)	14 (9.3%)	0.842	66 (8.7%)	7 (12.3%)	0.355

a Only applicable in cases without *H. pylori* in the current UGE.

mOLGA, modified Operative Link for Gastritis Assessment; GC, gastric cancer; UGE, upper gastrointestinal endoscopy.

### OLGIM staging

**Figure 3:**
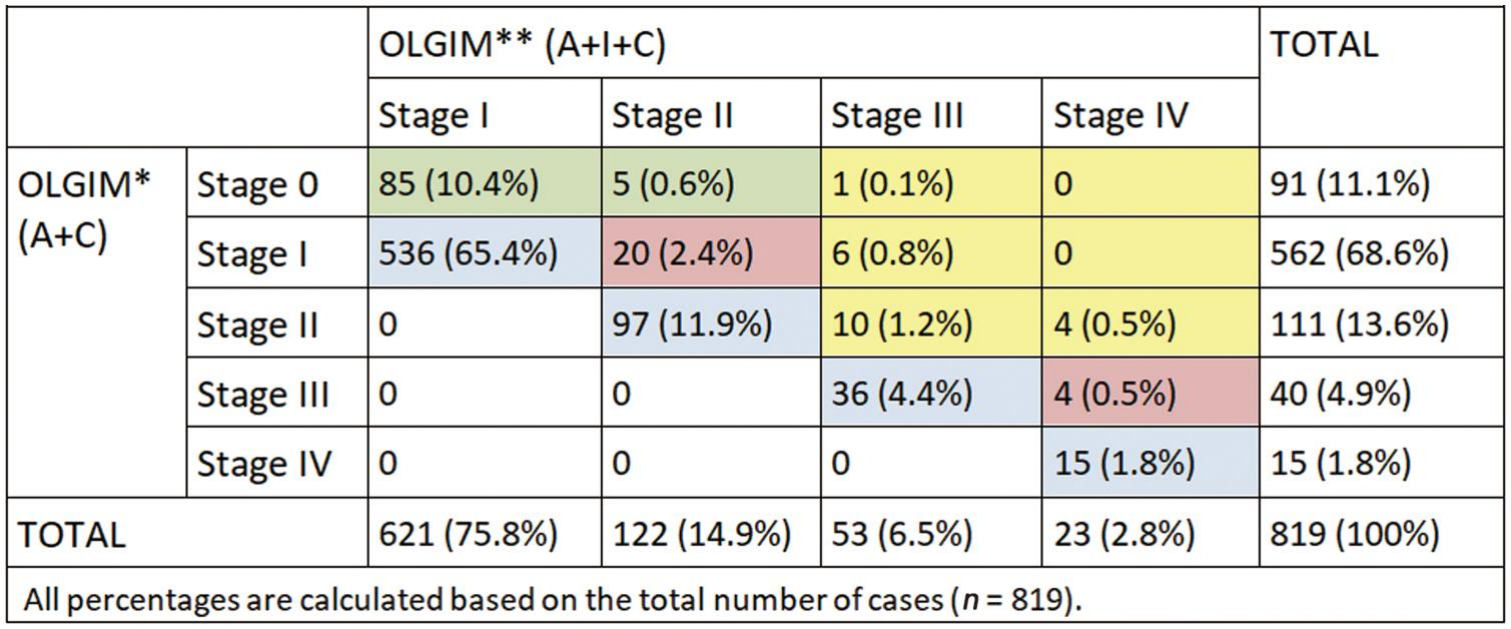
Comparison of OLGIM stage between using only biopsies from the antrum and the corpus and when adding incisura biopsy. *OLGIM using only antrum and corpus biopsies. **OLGIM using antrum, incisura and corpus biopsies. Pink indicates OLGIM upstaging only. Yellow indicates OLGIM upstaging that led to endoscopic surveillance recommendation. Green indicates OLGIM upstaging that, when associated with persistent *H. pylori* infection or a positive family history of GC, could lead to endoscopic surveillance recommendation. Blue indicates no changes in OLGIM stage when adding the incisura biopsy. OLGIM = Operative Link of Gastritis and Intestinal Metaplasia, A = antrum, I = incisura, C = corpus.

Similarly, OLGIM staging was assessed with and without the incisura biopsy. Without the incisura biopsy, the distribution of stages was 11.1% stage 0, 68.6% stage I, 13.6% stage II, 4.9% stage III, and 1.8% stage IV. After inclusion of the incisura biopsy, the distribution shifted to 0% stage 0, 75.8% stage I, 14.9% stage II, 6.5% stage III, and 2.8% stage IV, increasing the proportion of high-risk stages (III–IV) from 6.7% to 9.3% (a 2.6% increase). Overall, incorporating the incisura biopsy resulted in upstaging of OLGIM in 16.5% of cases (Fig. 3).

Patients with OLGIM upstaging were compared with those without changes in OLGIM stage. A higher proportion of upstaged cases originated from intermediate-risk (18.5% vs 8.8%, *P *= 0.002) and high-risk regions (0.7% vs 0.3%, *P *= 0.002). Alcohol consumption was also more frequent in patients with OLGIM upstaging, with current alcohol use reported in 18.5% vs 9.4% (*P *= 0.002), and previous alcohol use in 6.0% vs 3.5% (*P *= 0.002). In addition, active *H. pylori* infection was more frequent among patients with OLGIM upstaging (24.4% vs 13.7%, *P *= 0.002) (Table 3).

**Table 3:** Associated factors to change in OLGIM stage and follow-up of intestinal metaplasia—univariate and multivariate analysis

Variable	OLGIM stage					Follow-up of IM		
	No change (*N *= 684)	Upstage (*N *= 135)	Univariate (*P* value)	Multivariate (*P* value)	OR (95%CI)	No change (*N *= 798)	Change in follow-up (*N *= 21)	Univariate (*P* value)
Sex, *n* (%)			0.163					0.503
Male	270 (39.5%)	62 (45.9%)				322 (40.4%)	10 (47.6%)	
Female	414 (60.5%)	73 (54.1%)				476 (59.6%)	11 (52.4%)	
Age, *n* (%)			0.586					0.204
<67 years	327 (47.8%)	68 (50.4%)				382 (47.9%)	13 (61.9%)	
≥67 years	357 (52.2%)	67 (49.6%)				416 (52.1%)	8 (38.1%)	
GC risk by country of origin, *n* (%)			**0.002**					**0.021**
Low	622 (90.9%)	109 (80.8%)				716 (89.7%)	15 (71.4%)	
Intermediate	60 (8.8%)	25 (18.5%)		**<0.001**	2.53 (1.50-4.28)	79 (9.9%)	6 (28.6%)	
High	2 (0.3%)	1 (0.7%)		0.275		3 (0.4%)	0 (0%)	
Smoking habit, *n* (%)			0.422					0.492
Absent	399 (58.4%)	70 (51.9%)				455 (57.0%)	14 (66.7%)	
Previous exposure	172 (25.1%)	38 (28.1%)				206 (25.8%)	4 (19.0%)	
Current exposure	113 (16.5%)	26 (19.3%)				137 (17.2%)	2 (9.5%)	
Alcohol consumption, *n* (%)			**0.002**					0.296
Absent	596 (87.1%)	101 (74.8%)				681 (85.3%)	16 (76.2%)	
Previous exposure	24 (3.5%)	8 (6.0%)		0.058		32 (4.0%)	0 (0%)	
Current consumption	64 (9.4%)	25 (18.5%)		**0.002**	2.34 (1.38-3.91)	85 (10.7%)	4 (19.0%)	
Chromoendoscopy, *n* (%)	194 (28.4%)	36 (26.7%)	0.689			222 (27.8%)	8 (38.1%)	0.301
High-definition endoscopy, *n* (%)	684 (100%)	135 (100%)	–			798 (100%)	21 (100%)	–
Current *H. pylori* infection, *n* (%)	94 (13.7%)	33 (24.4%)	**0.002**	**0.004**	1.95 (1.23-3.10)	125 (15.7%)	2 (9.5%)	0.443
Previous *H. pylori*,^a^ *n* (%)	137 (20.0%)	28 (20.7%)	0.139			161 (20.2%)	4 (19.0%)	0.819
Visible lesion in incisura, *n* (%)	55 (8.0%)	18 (13.3%)	**0.049**	0.161		70 (8.8%)	3 (14.3%)	0.381

a Only applicable in cases without *H. pylori* in the current UGE.

OLGIM, Operative Link of Gastritis and Intestinal Metaplasia; IM, intestinal metaplasia; GC, gastric cancer; UGE, upper gastrointestinal endoscopy. Statistical significance is highlighted in the table with *P* values in bold.

On multivariate analysis, independent predictors of OLGIM upstaging were intermediate-risk origin (OR 2.53; 95% CI 1.5–4.28; *P *< 0.001), current alcohol consumption (OR 2.34; 95% CI 1.38–3.91; *P *= 0.002), and active *H. pylori* infection (OR 1.95; 95% CI 1.23–3.10; *P *= 0.004) (Table 3).

### Endoscopic surveillance recommendations

The inclusion of the incisura biopsy resulted in mOLGA upstaging from stages I–II to III–IV in 7.0% of patients, thereby leading to an indication for endoscopic surveillance in these patients according to MAPS III guideline [1]. No factors were significantly associated with recommendation for surveillance of gastric atrophy following the addition of the incisura biopsy (Table 2).

Regarding IM, OLGIM upstaging after inclusion of the incisura biopsy resulted in a recommendation for endoscopic surveillance in 2.6% of patients (Fig. 3), who otherwise would not have undergone follow-up. The only factor significantly associated with surveillance recommendation for IM due to the addition of incisura biopsy was GC risk based on country of origin. Patients requiring follow-up were more frequently from intermediate-risk countries than those not requiring follow-up (28.6% vs 9.9%, *P *= 0.021) (Table 3).

Finally, when considering both gastric atrophy and IM, 78 (9.6%) cases were recommended for endoscopic surveillance after inclusion of the incisura biopsy in mOLGA and OLGIM assessments. Four cases had overlapping recommendations, resulting in a total of 74 patients (9.0% of the cohort) receiving surveillance recommendation who would not have undergone follow-up without the inclusion of the incisura biopsy (Figs 2 and 3).

## Discussion

In this retrospective Spanish study, 819 patients with GCPL were included. In this cohort, the incisura biopsy identified cases of gastric atrophy, IM, and dysplasia that would otherwise have remained undetected (5.9%, 11.1% and 1.6%, respectively). This resulted in upstaging of mOLGA and OLGIM in a relevant proportion of patients (18.3% and 16.5%, respectively), leading to surveillance recommendations in 9.0% of the cohort.

Globally, GC remains one of the leading causes of cancer mortality due to its delayed diagnosis and poor prognosis at advanced stages. Accurate identification of GCPL and surveillance are therefore essential to reduce GC incidence and improve survival [4].

OLGA and OLGIM classifications were created to estimate the risk of developing GC and guide surveillance recommendations. These systems are based on the histological assessment of gastric biopsies obtained according to the updated Sydney protocol [5, 7, 11, 12]. Despite early evidence supported the inclusion of incisura biopsy in this protocol, its role has been debated in last years, and the recent MAPS III guideline considers its inclusion optional [1]. In addition, the original OLGA stage is not widely used in routine practice due to its complexity, as it requires quantification of the percentage of atrophy in each biopsy at each different location. In addition, atophy is often difficult to assess in non-oriented biopsies. Therefore, in most pathology reports, atrophy is described qualitatively (mild, moderate, or severe) rather than expressed as a percentage. Consequently, similar to OLGIM, OLGA staging in clinical practice is often estimated by gastroenterologists using these qualitative atrophy grades [13], and referred to as mOLGA in this study.

Our results are consistent with those of previous studies demonstrating the diagnostic relevance of incisura biopsy. Varbanova *et al*. [14] reported that sampling of the incisura identified 8% of gastric atrophy and nearly 3% of IM cases. Similarly, Eriksson *et al.* [15] found that incisura biopsy diagnosed 1.3% of gastric atrophy, 6% of IM and 0.4% of dysplasia. Another Brazilian study reported diagnostic rates of 2% of gastric atrophy, 2.3% of IM, and 0.6% of dysplasia [16].

Although the absolute proportion of lesions identified exclusively in the incisura appears relatively small, our data showed that 35.1% of all dysplasia cases were identified solely in this site. This finding reinforces the importance of incisura in detecting more advanced GCPL, and is consistent with previous observations suggesting that the incisura may harbour more severe lesions [8, 9].

Regarding risk stratification, several studies have demonstrated the influence of biopsy location on OLGA and OLGIM staging, similar to our findings. Isajevs *et al.* [8] reported that inclusion of the incisura biopsy led to upstaging in 18% of OLGA and 4% of OLGIM. Similarly, Varbanova *et al.* [14] observed significant decreases in staging when the incisura biopsy was excluded, while a Portuguese study reported downstaging of OLGA and OLGIM without the incisura biopsy in 8% and 22% of cases, respectively [17]. Furthermore, another study comparing biopsy site combinations on assessment of OLGA and OLGIM demonstrated that inclusion of the incisura biopsy significantly increased the detection of advanced GCPL and recommended its routine use to prevent the underestimation of OLGA/OLGIM [18]. On the other hand, a Russian study did not find statistically significant differences when adding the incisura biopsy in OLGA staging, although the heterogeneity of its inclusion criteria may partially explain this discrepancy [19].

Subsequently, clinical aspects were analysed to identify risk factors to upstage in mOLGA and OLGIM with the incisura biopsy. No variables were associated with changes in mOLGA staging or surveillance recommendations for gastric atrophy. In contrast, OLGIM upstaging was associated with current *H. pylori* infection, ongoing alcohol consumption, and origin from an intermediate-risk GC region. Surveillance recommendations for IM were also associated with intermediate-risk origin. The absence of an association with high-risk regions is likely explained by the small number of patients from high-risk origin, as it is well established that a higher prevalence of GCPL was observed in countries with high-risk of GC, when compared to low-intermediate risk regions [20].

Current *H. pylori* infection was associated with approximately a two-fold increase in the odds of OLGIM upstaging when the incisura biopsy was included. This finding is consistent with the well-established role of *H. pylori* infection in gastric carcinogenesis through the development and progression of GCPL [21]. Our findings support previous data: a Chinese study showed that *H. pylori* infection was an independent risk factor to advanced OLGA stages [22], similarly to Nam *et al*. [23], which also identified the infection as an independent risk factor for high-risk OLGA/OLGIM stages. Regarding alcohol, its relationship with GCPL remains controversial. Several studies have not identified alcohol as an independent risk factor for IM [24–26], whereas others have reported a dose–response association between alcohol consumption and the development of GCPL [27], and its link with aggravation of gastric atrophy over time [28]. The mechanisms underlying this association may involve alcohol-induced mucosal injury, including increased vascular permeability, oxidative stress, and local inflammation [29]. The differences between studies may reflect heterogeneity in alcohol exposure and dietary patterns, as well as differences in baseline GC risk across populations.

Additionally, incorporation of the incisura biopsy had measurable clinical implications. In our cohort, its inclusion resulted in surveillance recommendations in 9.0% of the patients who were upstaged to high-risk categories (mOLGA/OLGIM III-IV) and otherwise would not have been included in surveillance. Whether this magnitude of change justifies routine incisura biopsy in all clinical settings remains an important consideration. In intermediate- and high-risk populations, its systematical use may be justified, because it improves lesion detection and risk stratification. However, in low-risk regions or resource-limited environments, the incremental benefit should be balanced against potential increases in pathology workload and healthcare costs. Further studies evaluating the cost-effectiveness of routine incisura biopsy in different clinical settings are therefore warranted.

Importantly, the proportion of patients with surveillance recommendations observed in our study is likely a conservative estimate. According to the MAPS III guideline, additional factors such as family history of GC or persistent *H. pylori* infection may also influence surveillance recommendations in patients with low-risk OLGIM stages [1]. Because this information was not consistently available in our dataset, some patients who might qualify for surveillance under these criteria could not be fully identified. Therefore, the true impact of incisura biopsy on surveillance decision-making may be greater than the observed in this analysis.

This study has several limitations. First, its retrospective single-centre design may limit the generalizability of the findings. The cohort was derived from a tertiary centre from a low-risk GC region, and the study population was intentionally enriched with IM carriers. Consequently, the reported rates of OLGA and OLGIM upstaging and surveillance recommendations cannot be extrapolated to unselected endoscopic or screening populations. Future multicentre prospective studies are needed to validate these findings. Second, due to the retrospective design, some potentially relevant variables were not consistently available (such as family history of GC and detailed information on prior *H. pylori* eradication), which may have influenced the surveillance strategies and could have resulted in underestimation of the potential impact of incisura biopsy. Third, the study did not assess long-term clinical outcomes (such as lesion progression, GC incidence and survival). Therefore, prospective studies assessing follow-up are necessary to address whether the surveillance prompted by incisura biopsy translates into improved outcomes. Fourth, although biopsies were obtained according to routine practice based on the updated Sydney protocol, UGEs were performed by multiple endoscopists, across various settings, including inpatient and elective scheduled exams and not all UGEs were performed within the dedicated upper GI diseases agenda, so minor variations in biopsy technique may have occurred. Moreover, there was no registry about endoscopic classifications, such as Kimura–Takemoto for gastric atrophy or endoscopic grading of gastric IM. Finally, pathological assessment was not performed always by the same pathologist, but always was assessed by expert gastrointestinal pathologists; the type of IM (complete/incomplete) was not considered, but the OLGIM stage was assessed, and all the information for the mOLGA/OLGIM calculation was present in the pathology report.

Despite these limitations, the study presents several strengths. It includes a large cohort of patients with confirmed GCPL, ensuring a focused evaluation of the role of incisura biopsy in the assessment of GCPL, reducing possible selection bias. Additionally, the use of mOLGA reflects routine clinical practice, and allows to easily calculate the degree of atrophy from the information included in the pathology report, which would be missed in centres where the OLGA stage is not performed, and only the OLGIM is calculated. As far as we know, this is one of the first studies specifically assessing the impact of incisura biopsy on surveillance recommendations derived from mOLGA and OLGIM upstaging, providing objective data relevant to real-world clinical practice.

In conclusion, the addition of the incisura biopsy in carriers of GCPL results in upstaging of mOLGA and OLGIM classifications, which translates into surveillance recommendations in almost 10% of patients who otherwise would not have been followed. In addition, the incisura biopsy contributes to the diagnosis of more advanced GCPL, as more than one-third of all dysplasia cases were identified solely in this topography. Although the clinical relevance of routine incisura biopsy may vary across healthcare settings, our findings support its potential value for improving GC risk stratification and justify its inclusion in the assessment of GCPL, especially in patients from intermediate-risk of GC countries, with current alcohol intake and with persistent *H. pylori* infection. Considering carbon print, pooling incisura, and antral biopsies in a single vial may be considered.

## Supplementary Material

goag055_Supplementary_Data
