# Supplementary figures and images for: Impact of incisura biopsy on the surveillance of precursor lesions and gastric cancer risk assessment
Source: Gastroenterol Rep (Oxf). 2026 May 29;14:goag055. doi: 10.1093/gastro/goag055 (PMC13220145; doi:10.1093/gastro/goag055)

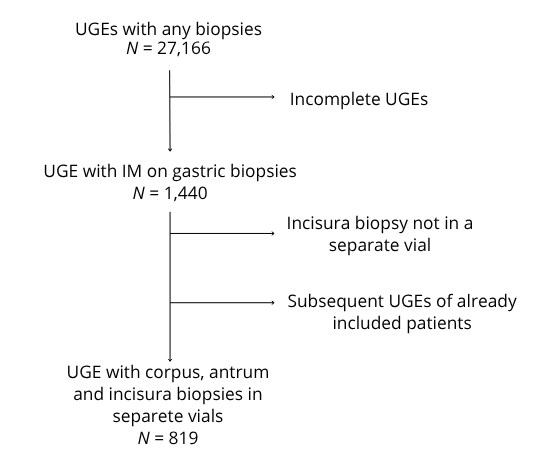

Supplement: goag055_Supplementary_Data [file goag055_supplementary_data.jpeg]
